# Clinic-to-Mechanism: Unraveling in-depth molecular dysfunctions caused by a GluN2B C-Terminal deletion in developmental and epileptic encephalopathies

**DOI:** 10.1038/s41398-026-04186-0

**Published:** 2026-06-24

**Authors:** Roza Szlendak, Nathalie Bouquier, Sylwia Rzońca-Niewczas, Annie Varrault, Tristan Bouschet, Mariola Rudzka-Dybała, Paulina Górka-Skoczylas, Philippe Lory, Dorota Hoffman-Zacharska, Julie Perroy

**Affiliations:** 1 IGF, Univ Montpellier, CNRS, Inserm, Montpellier, France; 2 Department of Medical Genetics, Institute of Mother and Child, Warsaw, Poland; 3 Clinic of Paediatric Neurology, Institute of Mother and Child, Warsaw, Poland

**Keywords:** Molecular neuroscience, Clinical genetics

## Abstract

Developmental and epileptic encephalopathies (DEEs) are severe neurodevelopmental disorders associated with genetic mutations, including some in GRIN genes encoding NMDA receptor (NMDA-R) subunits. Despite affecting the same receptor, each mutation may lead to distinct neurological disorders, emphasizing the necessity of understanding receptor dysfunction to tailor treatments effectively. In a genetic screen of DEEs patients, we identified a de novo pathogenic *GRIN2B* nonsense mutation, p.Glu839Ter (called GluN2B-E839*), which truncates the C-terminal domain (CTD) of the GluN2B subunit, a poorly characterized region critical for receptor function. This variant was prioritized for functional study due to the limited knowledge about the CTD’s role. We fully characterized the clinical presentation of the patient, who displayed intellectual disability, epilepsy, and hyperkinetic behavioral disorders. Molecular and cellular analyses in heterologous systems and patient-derived neurons revealed that GluN2B-E839* subunit assembles correctly with other subunits to form NMDA-Rs but exhibits reduced surface expression, impaired interactions with PSD95, and altered biophysical properties, including reduced current amplitudes, increased magnesium sensitivity, and diminished calcium influx. These dysfunctions likely contribute to impaired synaptic plasticity and DEEs pathophysiology. Our findings highlight the critical role of the GluN2B CTD in NMDA-R function and neuronal signaling and underscore the need for systematic characterization of *GRIN* variants to improve diagnostic precision and therapeutic targeting for DEEs. This study establishes a robust framework combining complementary experimental approaches with patient-derived preclinical models to link molecular dysfunctions to clinical phenotypes.

## Introduction

Developmental and Epileptic Encephalopathies (DEEs) are a group of severe neurodevelopmental disorders characterized by early-onset epilepsy, cognitive impairment, and often behavioral disturbances. DEEs encompass a diverse spectrum of genetic etiologies and recent advances in high-throughput sequencing have revealed a growing number of pathogenic variants within *GRIN* genes, encoding subunits of N-methyl-D-aspartate receptors (NMDA-Rs). The NMDA-Rs are vital voltage-dependent ionotropic glutamate receptors and ion channels, discovered over four decades ago [1, 2]. Their critical roles span neuronal development, synaptic plasticity, learning and memory, as well as excitotoxicity and other pathological processes [3–5]. NMDA-Rs are complex heterotetramers made up of two GluN1 subunits, binding glycine and two GluN2 (GluN2A-GluN2D) and/or GluN3 (GluN3A, GluN3B) subunits that bind glutamate and glycine, respectively. The diversity in subunit composition, coded by *GRIN1*, *GRIN2A-D*, and *GRIN3A-B* genes [6], imparts unique biophysical and pharmacological traits to these receptors [4, 7].

The unveiled numerous variants in *GRIN* genes have catalysed the creation of databases like the GRIN Portal (v1.0 - https://grin-portal.broadinstitute.org/). More than 700 pathogenic variants linked with neurodevelopmental disorders, including epilepsy and autism, have been identified [5, 8, 9]. Despite affecting the same receptor, each mutation may lead to distinct neurological disorders, emphasizing the necessity of understanding receptor dysfunction to tailor treatments effectively.

Disease-associated *GRIN* gene variants are found in all receptor domains and may alter protein expression and folding, receptor trafficking and localization, downstream signaling or biophysical properties of the receptor such as glutamate or glycine affinity, current density or magnesium sensitivity [6, 10]. While attention has traditionally focused on the well-structured regions of NMDA-Rs, the disordered C-terminal domain (CTD) has been relatively neglected due to its challenging nature, yet it is crucial for receptor function. The CTD is integral for interactions with proteins affecting NMDA-Rs’ biophysical and signaling attributes [7, 11]. Although variants in the CTD are substantial, about 20% of *GRIN* mutations identified via whole-exome sequencing occur here [12, 13], their role remains underexplored.

In this study, we identified a variant in the gene *GRIN2B* - c.2515 G > T (p.Glu839Ter, labelled as GluN2B-E839*) in a patient presenting intellectual disability, epilepsy, and hyperkinetic and behavioural disorders. This variant, causing the loss of the CTD of the GluN2B subunit, was classified as pathogenic. Using biosensors and electrophysiological recordings, we dissected this variant’s molecular dysfunctions in heterologous expression systems and patient-derived neurons, which are crucial models for understanding genetic contributions to disease [14]. Although the GluN2B-E839* variant forms heterotetrameric receptor with wild-type GluN1 and GluN2B subunits, it exhibits decreased expression at the cell surface, reduced interaction with scaffolding proteins like PSD95, and atypical biophysical properties compared to wild-type NMDA-Rs. Hence, not only did this study resolve a clinical case following an examination of the patient suffering from DEEs, but it highlights the functional importance of the previously underestimated CTD of the receptor. It also fulfils the need for a systematic and exhaustive approach to analyse *GRIN* mutations [10], by providing a comprehensive method to characterize the molecular and cellular dysfunctions induced by the mutation. Rather than limiting variant characterization to a simplistic binary classification, as in gain- or loss-of-function models, it is essential to understand the nature of the variants, their domain-specific localization, and their functional consequences, in order to enable accurate prognostic classification and personalized patient care.

## Material and methods

### Study design

Our study begins with a genetic investigation of new variants in DEEs patients, the selection of a variant of interest, followed by a detailed characterization of the patient’s symptoms associated with the variant and a comprehensive analysis of the molecular defects in the NMDA-R caused by the mutation. This includes studies in heterologous systems as well as assessments of cellular signaling defects in patient-derived neurons. This study design, intended as a comprehensive method to be systematically generalized for understanding the link between mutations and the dysfunctions leading to pathology, is summarized in Supplementary Fig. S1.

### Genetic investigation

The patient was referred to the Department of Medical Genetics of the Institute of Mother and Child (DMG IMC) in Warsaw for molecular testing using targeted next-generation sequencing (tNGS) employed for DEEs-related genes, using EIEE v.1/2016 panel (http://zgm.imid.med.pl/panele-ngs/). Libraries were sequenced on MiSeq sequencing platform using MiSeq Reagent Kit v3 (150-cycle) (Illumina, San Diego, CA, USA). Data analysis was performed using a data analysis pipeline developed within the IMC. The reads were aligned to the GRCh38 reference genome using BWA MEM. The characterization and classification of the identified variants was performed with ANNOVAR software using resources from genetic variants Genome Aggregation Database (gnomAD), prediction scores for non-synonymous variants using several widely used tools, PolyPhen2, Combined Annotation Dependent Depletion (CADD) and MutationTaster scores. The other software used to characterize and identify variants was Variant Studio v.3 (Illumina). All candidate variants were visually inspected using Integrative Genomics Viewer (Broad Institute). Sanger sequencing was used to confirm the presence of the identified *GRIN2B* classified as pathogenic variant and to perform segregation studies in family members.

### Patients clinical investigations

Clinical information from individual harbouring *GRIN2B* variant were provided by referring physician (paediatric neurologist). They included the patient’s personal history regarding progress of the pregnancy and childbirth, psychomotor milestone achievement, and neurological examinations. The history of epileptic seizures (type, age of onset, response to antiseizure medication), electroencephalographic data (background, epileptiform discharges, epileptic seizure if recorded), as well as the brain imaging data were also reviewed. Parents and/or legal guardians signed informed consent for the sample collection, genetic tests to be performed and genetic data to not only be used for diagnostic purposes.

### Plasmids

pRK5-HA-Venus-GluN2B and pRK5-HA-GluN1-4a DNAs were gifts from Dr Jean-Philippe Pin (Institut de Génomique Fonctionnelle). Nucleotide change c.2515 G > T (p.Glu839Ter) for the production of *GRIN2B* variant was achieved by PCR amplification of the GluN2B WT cDNA sequence with a forward primer containing an EcoRI restriction site upstream the ORF site (5’ ttctatcgattgaattccttt 3’) and a reverse primer containing a mutation generating a STOP codon and a SalI restriction site (5’ tttttgtcgacttagcagatgaaggtgatga 3’). The GluN2B WT sequence in pRK5 plasmid was then replaced with this PCR product by enzymatic digestion. For the BRET CODA-RET experiments, pRK5 plasmids were subcloned to encode Nluc-GluN1, V1-GluN2B, V2-GluN2B, V1-GluN2B-E839*, V2-GluN2B-E839* with V1 and V2 complementary non fluorescent halves of a Venus fluorophore. V1-CD8 and V2-CD8 (gift from Jonathan Javitch) were used as negative controls.

### Cell cultures and transfection

HEK293T cell lines were maintained at 37 °C in a humidity-controlled atmosphere with 5% CO_2_ in DMEM GlutaMAX^®^ medium (Gibco), supplemented with 10% fetal bovine serum, Penicillin-Streptomycin (100 U/ml, Gibco). Transient transfection of HEK 293T cells was achieved by the calcium phosphate method using CaCl_2_ 0.125 M and BES buffered saline (SIGMA). Transfections were carried out with a 1:3 ratio GluN1 : GluN2B subunits of the NMDA-R. In the heterozygous condition, transfections were performed using a 1:1 ratio of GluN2B WT to GluN2B-E839* subunits, while maintaining a constant 1:3 ratio of GluN1 to total GluN2B. Furthermore, D-2-amino-5-phosphonopentanoic acid (100 μM) (D-AP5, Hello Bio) and 7-chlorokynurenic acid (100 μM)(Tocris) were added to the medium to avoid excitotoxicity. All experiments were performed 24 h after transfection, under identical experimental conditions, using the same cell preparation, culture parameters, and transfection protocol; only the identity of the transfected plasmids differed between conditions.

### Human cortical neurons derived from patients’ fibroblasts

Fibroblast samples from patient with identified variant c.2515 G > T (p.Glu839Ter) in gene *GRIN2B* and healthy donor were obtained using the same collection protocol. A detailed characterization of the origin of the cell lines is presented in Supplemental Figure S2A. Human dermal fibroblast (HDF) line was established from skin punch biopsy from the same anatomical site using the same procedure, and both lines were reprogrammed and differentiated in parallel using identical protocols to minimize technical variability. HDF were reprogrammed in a complete xeno-free culture environment using non-modified RNAs (NM-RNAs) to generate iPSCs (StemRNA-NM reprogramming, Stemgent, USA). The HDF lines were cultured in Advanced DMEM/F-12 medium (Gibco) supplemented with 10% fetal bovine serum (Gibco), Glutamax (Gibco), and Antibiotic-Antimycotic agent (Gibco) at 37 °C in the incubator with 5% CO₂. The iPSCs were processed in parallel at the same time.

Two iPSC lines generated from somatic samples of patient and two from healthy control were maintained in feeder-free conditions using mTeSR Plus medium (StemCell) with Penicillin Streptomycin (100U/ml) on Matrigel (Corning) coated plates and maintained at 37 °C in humidified air with 5% CO₂, with the medium changed every 1-2 days. Cells were split as aggregates with Gentle Cell Dissociation Reagent (Stemcell) when reaching approximately 80% confluency (see Supplemental materials for pluripotency assessment). Selected characterized iPSCs clones were differentiated and then maturated in culture conditions as described by others [15] Neurons were maintained in Neurobasal supplemented with B27 (GIBCO) and 2 mM glutamine until the day of the experiment. Immunocytochemistry control using specific fluorescently-labelled antibodies (1:1000) was performed at different stages of differentiation (PAX6 (Biolegends), Nestin (StemCell), TBR1 (Cell Signaling), TUBB3 (Biolegends).

### Immunofluorescence analysis

Transiently transfected HEK 293T cells were washed in PBS and fixed with 4% paraformaldehyde (PFA) for 15–20 min at RT. Surface expression of NMDA-Rs was achieved by immunolabeling the extracellular tag – Venus - with the primary antibody (Anti-Green Fluorescent Protein Antibody, Sigma-Aldrich) for 1 h at RT under non-permeabilizing conditions. After washing, cells were incubated with secondary antibodies (anti-chicken IgG) conjugated to Alexa 594 fluorochromes (Molecular Probes) for 1 h at RT. After 3 further washing steps in PBS, Hoechst (Hoechst-33258, Sigma-Aldrich) (1:2000) was used for nuclear staining by incubation for 10 min at RT. Cells were mounted using Mounting Medium (Dako). Images were captured with an Axio Imager Z1 with ApoTome (Zeiss). The total amount of Venus-tagged GluN subunits was detected by the GFP endogenous fluorescent signal emitted by the Venus-GluN2B construct. A detailed list of antibodies is provided in Supplementary Table S1.

### Biotinylation experiments

Cells were washed twice with PBS + CaCl_2_/ MgCl_2_ and incubated with 0.25 mg/ml EZ-link sulfo-NHS-SS-Biotin (Pierce) for 30 min before two washes in Tris-HCl buffer to quench free reactive biotin. Cell lysis was then performed in lysis buffer (120 mM NaCl, 50 mM Tris HCl, pH 7.4, 2 mM EDTA, 1% Triton X-100) mixed with protease inhibitors, followed by incubation for 30 min at 4 °C. Samples were centrifuged at 16,000 g for 15 min at 4 °C, and the protein concentration of the supernatants was determined using a BCA kit (Sigma). A fraction of the lysate was kept as a whole input (total) reference sample. Biotinylated proteins were purified by pulling down on Neutradivin-coated magnetic beads (Spherotech) for 1 h. Beads were subsequently washed 3 times with lysis buffer, and biotinylated proteins were eluted in 4X SDS-PAGE denaturant buffer (Bio-Rad) supplemented with 10% β-mercaptoethanol and heated for 5 min at 60 °C. Total and surface protein expression was analyzed by Western Blot. Samples were loaded on 4–15% gradient acrylamide gels and then transferred onto nitrocellulose membranes (Amersham). Membranes were incubated in blocking buffer (PBS, 0.1% Tween-20, and 5% skimmed dried milk) for 1 h at room temperature and then with rabbit anti-GFP antibody (Invitrogen) primary antibodies in blocking buffer. They were washed and incubated with HRP-conjugated secondary antibody (1:5000 in blocking buffer). Immunoreactivity was detected with an enhanced chemiluminescence method (ECL detection reagent, GE Healthcare). After stripping of the first antibody labelling (0.5% sodium azide), membranes were re-blotted with mouse anti-HA (Covance) for GluN1 expression or mouse anti-actin (Sigma) for loading control. Immunoreactive bands were quantified by densitometry using the Image J software.

### Co-immunoprecipitation experiments

HEK239T cells were transfected with plasmids encoding HA-GluN1, HA-GluN2B and PSD95-GFP (ratio 1:3:6 for GluN2B WT or GluN2B-E839* homozygous conditions and 1:1.5-1.5:6 for GluN2B WT/839* heterozygous conditions) for 24 h. After two washes in PBS, cells were solubilized in a buffer containing 50 mM Tris-HCl pH 7.4, 150 mM NaCl, 0.5 mM EDTA, 1% (v/v) Triton and a protease inhibitor cocktail (Roche). Equal amount of proteins were incubated with GFP-Trap beads (Chromotek) overnight at 4 °C. Beads were washed three times with lysis buffer before elution in 4X Laemmli Sample buffer (Bio-Rad) supplemented with 10% β-mercaptoethanol and heated for 5 min at 60 °C. Samples were analyzed by Western blotting (see biotinylation section, with first anti-HA antibody, then stripping and re-blotting with anti-GFP and anti-actin antibodies).

### Single-cell BRET imaging

Single-cell BRET imaging experiments were performed and analyzed as previously described (Goyet et al., 2016). Briefly, cells were seeded in 35 mm diameter glass dishes (MatTek®) coated with poly-ornithine. Just before BRET/CODA-RET recordings, the culture medium was replaced by a recording medium (NaCl 140 mM, CaCl_2_ 2 mM, KCl 3 mM, HEPES 10 mM, D-glucose 10 mM, pH7.4, 330 mOsm). Glycine (10 μM; SIGMA), was added to the medium before the experiment. All images were obtained using a bioluminescence-dedicated inverted fluorescence microscope (Axiovert 200 M; Carl Zeiss) with a Plan Apochromat 63×/1.40 oil M27 objective at RT and collected with an Evolve camera (Photometrics) equipped with a Neofluar 40X/1.3 planar EC oil objective (M27, ZEISS) and an ORCA-Quest qCMOS camera (Hamamatsu), controlled by Metamorph software (Molecular Devices). Sequential acquisitions of light emission at 460 nm and 535 nm wavelengths (Em460 and Em535) were performed at 5 MHz for the indicated acquisition times: Nluc: 20 sec, YFP: 40 sec, 5 min after the addition of 50 µM furimazine. Images were analyzed using the BRET Analyzer macro on Fiji [16] Overall, this analysis uses an open-source toolkit for Fiji (https://github.com/ychastagnier/BRET-Analyzer) performing four key steps in the analysis: (1) image background subtraction, (2) image alignment over time, (3) a composite thresholding method of the image, and (4) pixel by pixel division of the image and distribution of the ratio intensity on a pseudocolor scale. The 535 nm/460 nm ratio images were generated by pixel-by-pixel division of the absolute light intensities at 535 nm and 460 nm. Results were analyzed by nonlinear regression on a pooled data set from 3 independent experiments.

### Electrophysiological recordings

All experiments were performed in the whole cell configuration of the patch-clamp technique. HEK293T cells, seeded in 35 mm diameter plastic dishes at low confluency (1 × 10^4^ cells) were co-transfected with pRK5-HA-GluN1 and pRK5-HA-Venus-GluN2B and/or pRK5-HA-Venus-GluN2B-E839* plasmids and were targeted by fluorescence microscopy. Measurements were performed with pipettes of 4–5 MΩ resistance when filled with the appropriate intracellular medium (pH of 7.2 and an osmolarity of 300 mOsm; EGTA 20 mM, CaCl_2_ 0,5 mM, CsCl 140 mM, HEPES 10 mM, D-glucose 10 mM, ATP-Na2 2 mM). The cells were perfused continuously at RT with extra-cellular physiological bath solution with a pH of 7.4 and an osmolarity of 330 mOsm (NaCl 140 mM, CaCl_2_ 2 mM, KCl 3 mM, HEPES 10 mM, D-glucose 10 mM). Glycine (10 μM; Sigma) was added to the medium before the experiment. Whole­cell currents were recorded from cells continuously perfused using a fast gravity perfusion system. Successive 20 sec-applications of NMDA-R agonist (NMDA 100 µM) were performed with/without inhibitor (MgCl_2_ 1 mM), spaced with external medium rinses for 60 sec. Currents were recorded using an Axopatch 200B amplifier (Axon Instruments) and digitized at 3 kHz. The data were then analyzed using the Clampfit 11.2 software from Axon Instruments (Molecular Devices). For human iPSC-derived neurons, the excitability was estimated in the current clamp mode for either spontaneous or evoked firing activities. Switching to the voltage-clamp mode was done for each recorded neurons to measure the presence voltage-gated sodium current.

### Ca^2+^ measurements

On day 52 of the neuronal differentiation, cells were transduced with GCamp6s-P2A-Scarlet lentiviral particles allowing the expression of the calcium indicator and a fluorescent protein, and in vitro calcium imaging experiments were performed from days 62 to 70. Just before the calcium imaging recordings, the culture medium was replaced by an extracellular medium (NaCl 140 mM, CaCl_2_ 2 mM, KCl 3 mM, HEPES 10 mM, D-glucose 10 mM, glycine 10 μM) with a pH of 7.4 and an osmolarity of 330 mOsm. Calcium imaging was recorded from cells continuously perfused using a fast gravity perfusion system. NMDA (100 µM) application for 60 sec, alone or with antagonists, ifenprodil (100 nM) or zinc (50 nM) was spaced with appropriate medium (without NMDA) rinses. All images were obtained using an Axio Observer 7 KMAT fluorescence microscope (Carl Zeiss), equipped with a Plan-Neofluar 40x/1.30 EC oil objective (M27, ZEISS®), Exc 470/40 nm – Em 525/50 nm filters and ORCA-Quest qCMOS camera (Hamamatsu); all controlled with Metamorph software.

Analysis was performed using an opensource toolset for Fiji (custom-written code CalciumExtractTracesAndTimings designed by Yan Chastagnier). The code extracts the mean intensity value of regions of interest (ROI) drawn on 4–12 cells randomly chosen per field of view, for each image in the time series. Ca^2+^ fluctuations from ROIs were reported as the relative fluorescence changes ΔF/F0 = (F − F_0_)/F_0_, where F is the signal measured over the time of recording and F0 is the basal fluorescent intensity before drug application. The mean +/- SEM of the maximal fluorescence fluctuation after drug application was calculated on ROIs drawn on the cells: 4–12 ROIs per field of observation, from 1 to 5 fields per dish, in 14 to 17 dishes from 2 clones per condition, in control and mutated cell lines from 3 independent differentiations.

### Statistical data analysis

Analyses were performed using GraphPad Prism 7 (GraphPad Software). Statistical analyses were performed with the non-parametric Mann and Whitney test to compare two independent samples or Kruskal–Wallis test for more than two independent samples. Values shown are mean ± standard error of the mean (SEM) of at least 3 independent experiments. All detailed information regarding the statistical analyses is presented in Supplementary Table S2.

## Results

### Identification of a heterozygous GRIN2B mutation p.Glu839Ter (GluN2B-E839*), deleting the CTD of the NMDA-R subunit

**Fig. 1:**
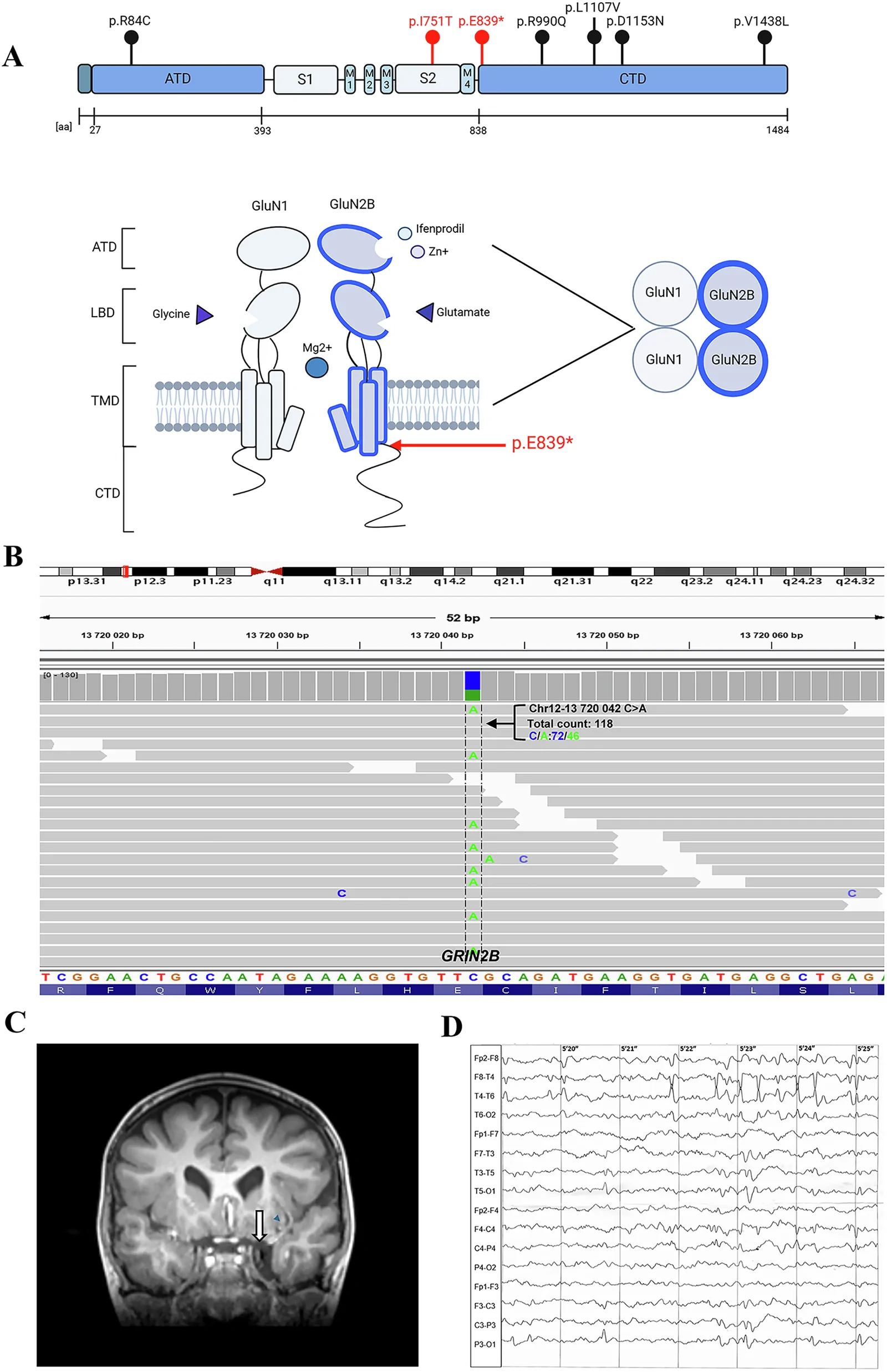
p.Glu839Ter (GluN2B-E839*), a nonsense mutation of *GRIN2B* in a DEEs patient. **– (A)** Distribution of identified *GRIN2B* variants along the GluN2B protein sequence. Color code: pathogenic variants in red; likely pathogenic and variants of uncertain significance (VUS) in black. GluN2B domains include the amino-terminal domain (ATD), S1 and S2 which form the ligand-binding domain (LBD); transmembrane spanning domains (M1-M4;TMD) and C-terminal domain (CTD). The p.Glu839Ter mutation (p.E839*) location is marked by a red arrow. It leads to the insertion of a STOP codon, suppressing the CTD of GluN2B. Figure made in BioRender.com. **(B)** Aligned reads from sequencing loaded into Integrative Genomics Viewers (IGV) in the BAM format. The sequence on the bottom represents the reference genome. Note that the single base substitution at position g.13720042 of Chromosome 12 is shown as a C → A change because *GRIN2B* is coded on the antisense strand. IGV displays the sequence of bases as they appear in the FASTA file for the reference genome - reverse one for *GRIN2B* gene. **(C)** Patient’s MRI image at 6 years showed an arachnoid cyst in the middle fossa on the left side with a linear dimension of 36 mm (white arrow). **(D)** EEG recordings at 8 years old. The focal changes in the EEG are located in the temporal and central parietal-occipital areas bilaterally, sharp waves have the highest amplitude over the right temporal.

Genetic diagnostics for patients referred to the Department of Medical Genetics of the Institute of Mother and Child, clinically diagnosed as epileptic encephalopathy, DEEs or drug-resistant epilepsy, were performed using targeted next-generation sequencing (tNGS). The gene panel included 48 genes identified in 2016 as implicated in DEEs syndromes, based on the Online Mendelian Inheritance in Man (OMIM) database (Supp. Table S3). The full genetic analysis will be released in a dedicated publication. Briefly, the analysis covered coding exons, exon/intron boundaries, and splice regions (±10 bp). Each of the 694 patients analysed generated a dataset describing gene variants deviating from reference sequences. 339 patients had pathogenic, likely pathogenic, or variants of uncertain significance (VUS), with 409 variants identified in total. Among these, 21 variants were found in *GRIN* genes: 14 variants classified as VUS, 3 as likely pathogenic, and 4 as pathogenic. Of the 4 pathogenic variants, 2 were identified in the *GRIN2B* gene (Fig. 1A). We decided to focus on one of them, located just upstream of the coding sequence of the CTD, theoretically leading to its deletion, because this region known as the most variable is the least characterized.

Hence, we considered the heterozygous *GRIN2B* variant (NM_000834.5): c.2515 G > T (p.Glu839Ter) as the candidate associated with the patient’s phenotype (Fig. 1B). Analysis of parents DNA by Sanger sequencing revealed that this mutation was de novo. Located in exon 13 of 14 in *GRIN2B*, the p.Glu839Ter variant results in a truncated GluN2B subunit of the NMDA-R, lacking the entire cytosolic CTD (Fig. 1A). In silico analysis using MutationTaster further classified the variant as disease-causing, with a CADD pathogenicity score of 43. Thus, we classified this nonsense variant as pathogenic according to the criteria set forth by the American College of Medical Genetics and Genomics (ACMG). We will refer to this variant as GluN2B-E839* throughout the text and denote it as 839* in the figures.

### Clinical features of the GluN2B-E839* patient

The proband was a Polish girl with intellectual disability, epilepsy, and hyperkinetic and behavioural disorders. She was an only child of healthy non-consanguineous parents. There was no burden for epilepsy nor neurological disease in the family history. Pregnancy was complicated by arterial hypertension during the eighth month. The newborn’s condition was good, with an Apgar score of 10/10, weight was 3430 g, length 53 cm, and head circumference 36 cm. Transcranial ultrasonography revealed no brain abnormality. The neonatal period was free of non-neurodevelopmental complication. However, the patient’s psychomotor development was delayed in all areas from the first days on. Muscle tone was reduced during infancy. The patient acquired independent walking at the age of 20 months. Her speech development was also delayed. She spoke single words around the age of 3 years. She made slow progress during rehabilitation and speech therapy. Currently, she performs at the lower limit of intellectual disability to a mild degree with hyperkinetic disorder. Autism spectrum disorders have been ruled out. Metabolic disorders were excluded as part of the diagnosis (Tandem MS, GCMS, SAICAR normal results). Two MRI examinations at 3 and 6 years old showed an arachnoid cyst in the middle fossa on the left side with a linear dimension of 36 mm (Fig. 1C). Echocardiography revealed no abnormality, renal ultrasound showed features of duplicated pyelocalyceal system in the right kidney. The patient has postural defect-flat feet, kyphosis. Neurological examination showed reduced muscle tone, ligamentous laxity, impaired visuomotor coordination and dyspraxia, especially in the upper limbs. Due to neurodevelopmental disorders, scalp electroencephalography (EEG) was performed repeatedly during sleep. Results were abnormal. Initially, a predomination of generalised paroxysmal changes in the form of 0.5–6 s spike discharges and spike-wave and slow-wave complexes at 2–5 Hz was observed. At the age of 3, myoclonus in the limbs occurring during the discharges was recorded on the video EEG. Absence seizures were also observed at this time. Treatment was initiated when the girl was 4.5 years old. The patient was initially treated with Depakine chronosphera (VPA), and gradual resolution of myoclonic sleep-induced seizures and absence seizures was achieved. EEG recordings repeatedly showed generalised paroxysmal changes in the form of a 2–3 s, series of slow wave discharges mixed with acute slow wave, spike-wave complexes, and changes localised in the temporal area bilaterally, sometimes continuous changes localised in the temporal and central parietal-occipital areas bilaterally, nearly continuously above the right temporal area (Fig. 1D). Depakine was combined with Clobazam (CLB) and a gradual improvement of the recordings was obtained, ictal activities were limited to those located in the temporal-parietal area, different on each side. At the age of 12, a normalisation of the recordings was obtained. Depakine was gradually discontinued due to puberty. Attempted treatment with levetiracetam was terminated due to exacerbation of hyperkinetic and behavioural disorders. Currently, she does not take any anti-epileptic medication. Due to behavioural disorders and hyperactivity, she has been treated by a psychiatrist, initially with Risperidone, without much effect, then with aripiprazole with good results.

### Assessment of GluN2B-E839* molecular dysfunctions in a heterologous expression system

To characterize the molecular dysfunctions induced by the GluN2B-E839* variant, we replicated the mutation in a plasmid and expressed it via transient transfection in HEK293T cells, which do not endogenously express GluN subunits. This heterologous expression system allows for the isolation or combination of wild-type and/or mutant subunits, enabling controlled studies of their differences. We can thus dissect the molecular dysfunctions of NMDA receptors caused by the absence of the GluN2B C-terminal domain by comparing the homozygous condition (GluN1/ GluN1 /GluN2B-E839* / GluN2B-E839*) with wild-type receptors (GluN1 / GluN1 / GluN2B WT / GluN2B WT), but also—and most importantly—investigate the function of receptors heterozygous for the mutation, which mimics the clinical condition: (GluN1 / GluN1 / GluN2B WT / GluN2B-E839*). In this model, we examined subunit associations with each other and scaffolding proteins, as well as receptor cell-surface expression and function.

#### The GluN2B-E839* variant interacts with both wild-type GluN1 and GluN2B subunits

**Fig. 2:**
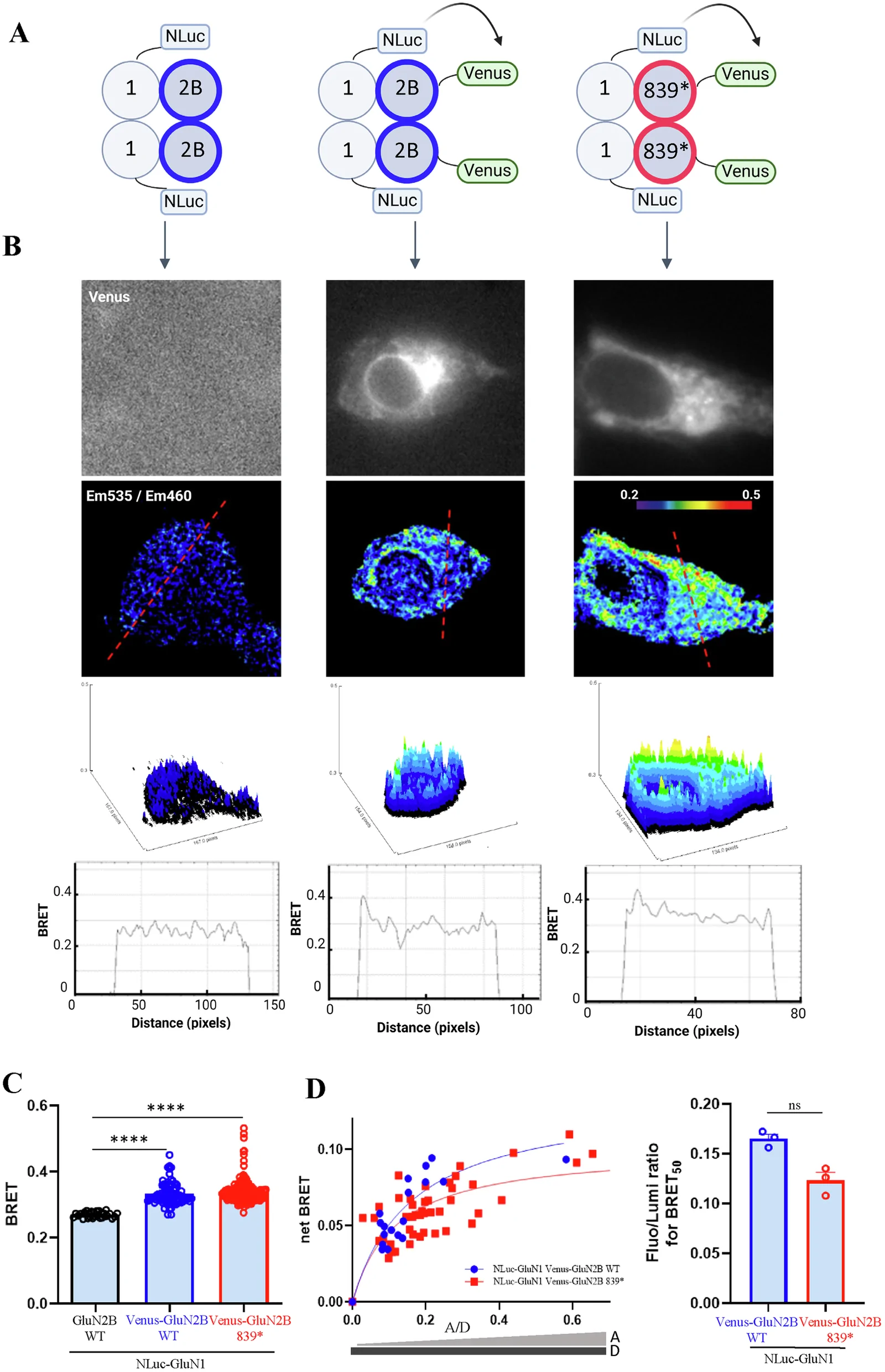

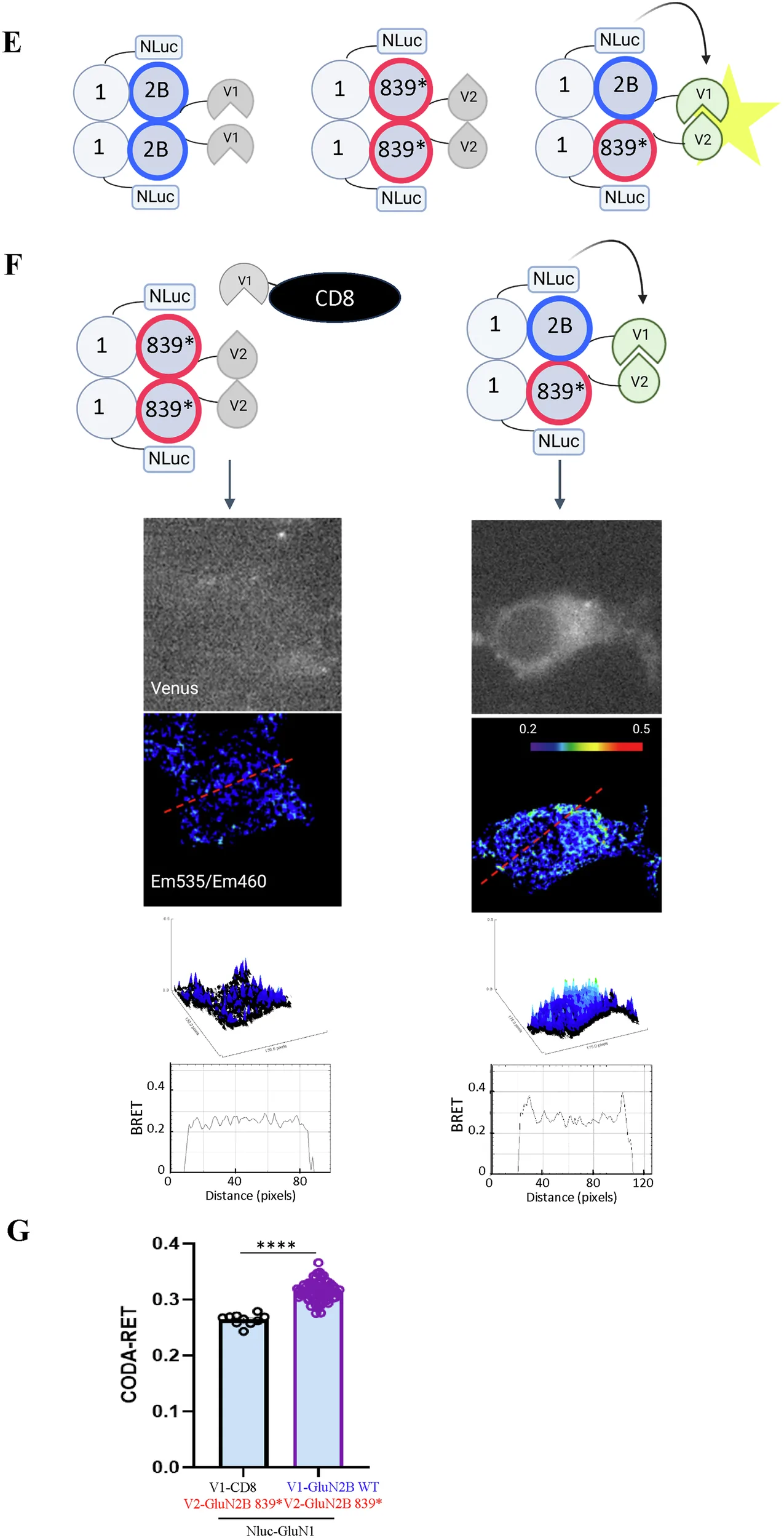
GluN2B WT and GluN2B-E839* display similar affinities for GluN1, and the three subunits can assemble into NMDA-Rs. **(A)** Scheme of constructs expressed in HEK cells to assess interactions by BRET. **(B)** and **(C)**- representative illustration (B) and quantification (C) of BRET imaging recorded in cells expressing NLuc-GluN1 with untagged GluN2B (basal BRET, left column) or with Venus-GluN2B (middle column) or Venus-GluN2B-E839* (right column) subunits. In (B) each column displays the expression of the acceptor (Venus), the BRET signal obtained by pixel-by-pixel division of the light emitted by the acceptor over the light emitted by the donor (Em 535 / Em 460) initiated by catalytic oxidation of NLuc substrate, a 3D illustration of the BRET (Fiji, Surface Plot), and a plot profile of BRET intensities along a line crossing the cell (red dashed line). (C) Scatter plot representing the mean ± SEM of BRET intensities measured in regions of interest (ROI) drawn on cells from 3 experiments. Kruskal-Wallis test p < 0.0001. Dunn’s multiple comparisons: **** p < 0.0001. **(D)** Left panel: Titration curves obtained by expressing BRET signals as a function of the acceptor A/donor D expression ratio. Saturation of the BRET signal when all donor is linked to the acceptor reports specific interaction between subunits. Curves were generated by pooling data from three independent experiments and fitted by nonlinear regression using a one-site binding hyperbola model (GraphPad Prism 7). (D) Right panel: Histogram showing the apparent affinity (Kd) values derived from individual titration curves fitted separately for each experiment. Data are presented as mean ± SEM. The Kd corresponds to the A/D ratio required to reach 50% of the maximal BRET signal. **E** Principle of CODA-RET, combining complementation of Venus acceptor and BRET. Providing interaction of proteins fused to V1 and V2 non-fluorescent moieties of Venus, proximity and complementation between V1 and V2 refolds a fluorescent Venus, which will be the acceptor of BRET. Note that due to the lack of fluorescence in the absence of complementation, possible other combinations of subunits (like V1-GluN2B/V1-GluN2B, left or V2-GluN2B-E839*/V2-GluN2B-E839*, middle) are not reported by this assay. Hence, subsequent BRET between NLuc-GluN1 and complemented V1-V2 exclusively highlights the existence of complexes containing the 3 types of subunits. **(F)** and **(G)**- same legend as in 2B and C, except that “Venus” images here report the V1- V2 complementation. Mann-Whitney test **** p < 0.0001 (GraphPad Prism 7).

Functional NMDA-Rs are obligate heterotetramers [17]. Using Bioluminescence Resonance Energy Transfer (BRET, Fig. 2A), we measured the ability of the mutated GluN2B-E839* subunit, compared to wild-type, to bind the GluN1 subunit (Fig. 2B). Cells expressing Nluc-GluN1 subunit and Venus- GluN2B-E839* or Venus-GluN2B displayed a significantly higher BRET signal than the basal signal recorded in cells expressing NLuc-GluN1 and untagged GluN2B (Fig. 2C). The hyperbolic increase of the BRET signals recorded in each cell as a function of acceptor/donor expression ratio (A/D) provided evidence for specific interactions of Nluc-GluN1 with both Venus-GluN2B or Venus- GluN2B-E839* (Fig. 2D) (while a random collision between proteins would have generated a linear increase of BRET without saturation). Furthermore, the relative affinity of the mutated GluN2B-E839* subunit for GluN1 subunit (Kd= 0.12 + 0.008), quantified by the A/D ratio required to get 50% of the maximal BRET signal, was similar to the relative affinity of GluN2B subunit for GluN1 subunit (Kd= 0.16 + 0.004).

Since our patient is heterozygous, we studied the potency of GluN1, GluN2B WT and GluN2B-E839* to interact within the same complex. The Complemented Donor-Acceptor Resonance Energy Transfer (CODA-RET) method is well-suited for addressing this question (Fig. 2E): non-fluorescent moieties of Venus, V1 and V2, can be refolded into a fluorescent tag by complementation, providing that the two proteins they are fused to interact. This complemented Venus serves as the fluorescent acceptor of BRET if a third, NLuc-fused, protein also interacts. Playing with subunit combinations, we found BRET signals between NLuc-GluN1 and complemented V1-GluN2B WT and V2-GluN2B-E839* (Fig. 2F). Indeed, Venus fluorescence in cells transfected with V1-GluN2B WT and V2-GluN2B-E839* revealed that the mutated and wild-type subunits of GluN2B could be found in the same complexes. By opposition, no complementation was found between V1-CD8 membrane protein (used as a negative control of complementation) and V2-GluN2B-E839* (Fig. 2G). The complemented Venus from V1-GluN2B WT / V2-GluN2B-E839* interaction displayed significant BRET with Nluc-GluN1 (Fig. 2G), highlighting the interaction of GluN1, GluN2B WT and GluN2B-E839* subunits in the same NMDA-R.

#### The GluN2B-E839* variant reduces surface trafficking of NMDA-R

**Fig. 3:**
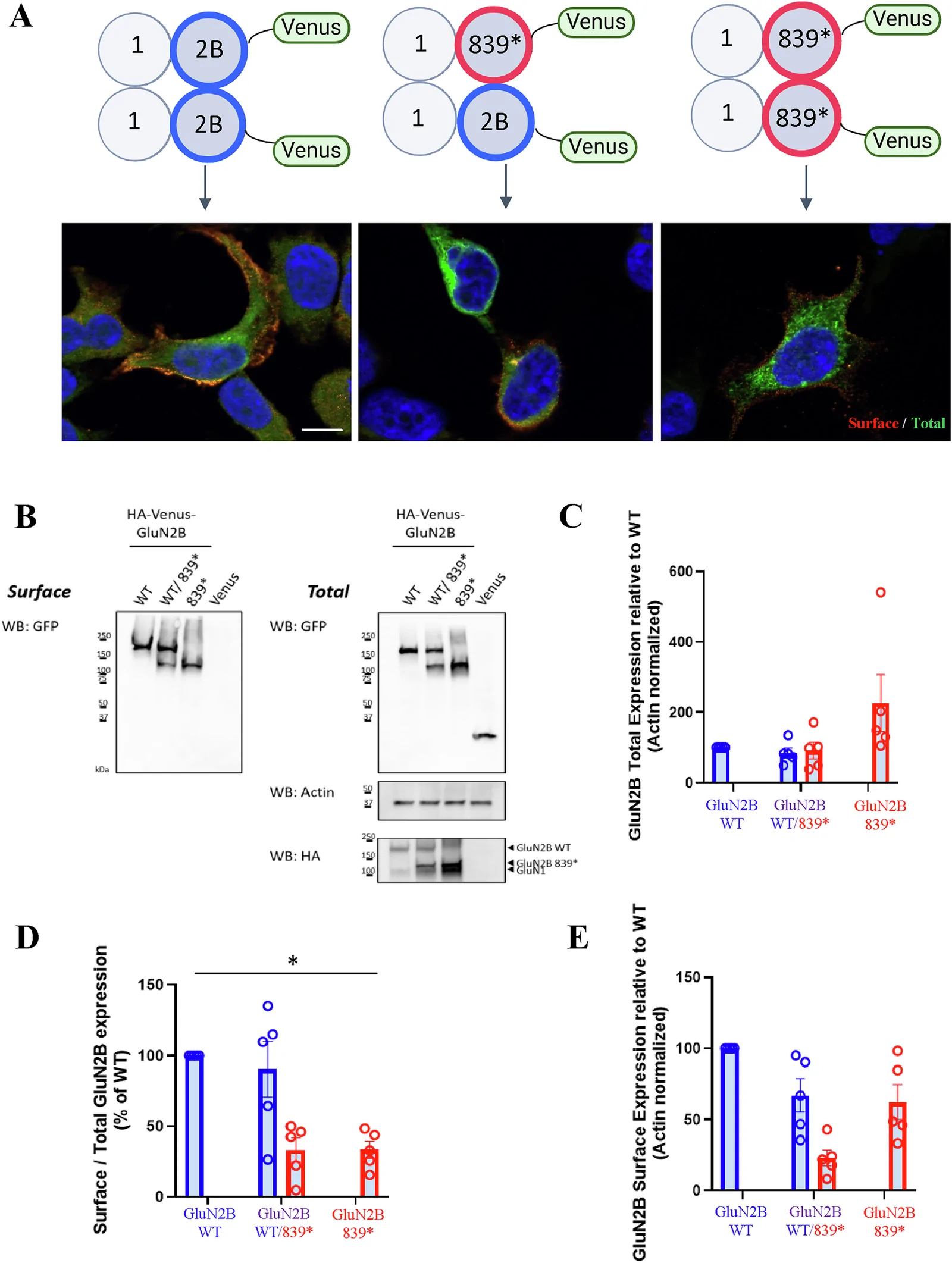
Analysis of NMDAR surface expression in transfected HEK 293T cells. **(A)** Scheme of constructs expressed in HEK 293T cells. Note that other compositions of NMDA-R are possible in the heterozygous condition: GluN1/GluN2B or GluN1/GluN2B-E839*. Total (Venus fluorescence - Green) and cell surface expression (red Ab raised against extracellular Venus tag) of Venus-GluN2B subunits are stained. Scale bar: 10 μm. **(B-E)** Representative Western Blot **(B)** and quantification **(C-E)** of NMDA-R surface expression by biotinylation experiments. Cells were transfected with HA-GluN1 together with HA-Venus-GluN2B and /or HA-Venus-GluN2B-E839* and incubated with biotin to label surface proteins. The biotinylated fraction pulled down by neutravidin beads (Surface, left) and the total proteins (right) were measured with an anti-GFP detecting Venus expression. **(C-E)** Bar graphs representing the mean ± SEM of GluN2B total **(C)**, surface/total **(D)** or surface **(E)** expression relative to WT (GluN2B/Actin ratio). Results were analyzed on a data set from 5 independent experiments. Kruskal-Wallis test p < 0.05 followed by Dunn’s multiple comparisons test *p < 0.05 (comparison to GluN2B WT expression).

To study the functional consequences of the GluN2B-E839* variant, we studied the NMDA-R expression and its cell surface targeting. Immunofluorescence analysis of HEK 293T cells expressing GluN1, Venus-GluN2B WT and/or Venus-GluN2B-E839* (Fig. 3A) enables to report the total expression using Venus fluorescence while the surface expression was imaged by immunolabeling of the extracellular Venus tag using a red-coupled, anti-GFP primary antibody under non-permeabilizing conditions. GluN2B-E839*-containing NMDA-Rs showed reduced surface expression compared to the wild-type, and this reduction was even more pronounced in the homozygous state than in the heterozygous state. We further quantified these differences by measuring both the total levels of expression and the cell surface subunits (Fig. 3B–E), by biotinylation experiments. Although this difference did not reach statistical significance, the total expression level of GluN2B-E839* subunits tended to be higher (approximately 2.25-fold) than that of wild-type GluN2B subunits when expressed separately (Fig. 3C); in contrast, the proportion of receptors delivered to the membrane was significantly reduced for GluN2B-E839* (Fig. 3D), resulting in a slight decrease in surface expression of GluN2B-E839* compared to the wild-type subunit (Fig. 3E). Consistently, the heterozygous condition showed intermediate levels of total (Fig. 3C) and surface (Fig. 3E) expression. Interestingly, when co-expressed, the proportion of total wild-type GluN2B subunits reaching the cell surface (Fig. 3D) and the amount expressed at the cell surface (Fig. 3E) was higher than the GluN2B-E839* subunits suggesting a heterogeneous composition of NMDA-Rs in subunits, some containing only GluN2B wild-type subunits. Taken together, these data show a reduced expression of the GluN2B-E839* at the cell surface despite its higher total expression and suggest impaired cell-surface trafficking of GluN2B-E839*-containing NMDA-Rs.

#### The GluN2B-E839* variant interacts less than GluN2B with PSD95

**Fig. 4:**
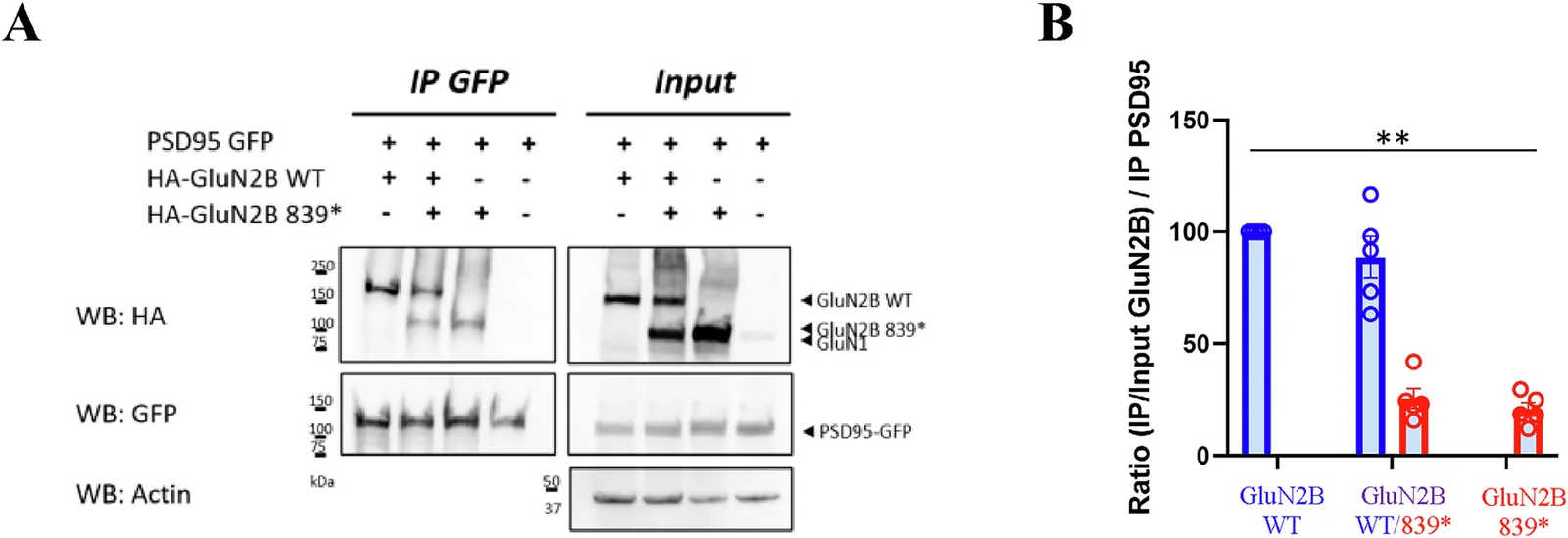
GluN2B CTD deletion decreases NMDA-R interaction with PSD95. **(A)** Co-immunoprecipitation of GluN2B and PSD95: HEK-293T cells were transfected with plasmids encoding PSD95-GFP and HA-GluN1 with HA-GluN2B WT and/or E839*. Proteins were immunoprecipitated with GFP-Trap® beads. GluN2B and PSD95 expressions in immunoprecipitates and inputs were analyzed by Western blotting using anti-HA and anti-GFP antibodies. Actin was also blotted as a loading control. **(B)** Quantification of the amount of GluN2B/PSD95 binding (Ratio [(IP/ Input) GluN2B / IP PSD95]). Bar graphs represent the mean ± SEM relative to WT. Results were obtained by densitometry analyses of blots from five independent experiments performed on different sets of cultured cells. Kruskal-Wallis test p < 0.01 followed by Dunn’s multiple comparisons test ** p < 0.01 (comparison to GluN2B WT expression).

PSD95 is a scaffolding protein that plays a critical role in the surface trafficking of NMDA-Rs. By binding to the CTD of NMDA-R subunits, PSD95 stabilizes the receptors at the postsynaptic membrane [18]. We studied the ability of the wild-type GluN2B subunit and GluN2B-E839* variant to interact with PSD95 by co-immunoprecipitation, using GFP-TRAP immunoprecipitation (Fig. 4). The fraction of HA-tagged GluN2B-E839* subunits co-immunoprecipitated with PSD95-GFP was strongly decreased compared to HA-GluN2B WT. Of note, while strongly reduced, GluN2B-E839* interaction with PSD95 was not totally abolished in the homozygous conditions, suggesting possible unknown low-affinity PSD95-binding sites in other domains of the CTD-deleted subunit. Thus, given the essential role of PSD95 in NMDA receptor synaptic localization and function, the impaired interaction between GluN2B-E839* and PSD95 is likely to promote functional deficits of the mutant receptor.

#### The GluN2B-E839* variant alters NMDA-R functions

**Fig. 5:**
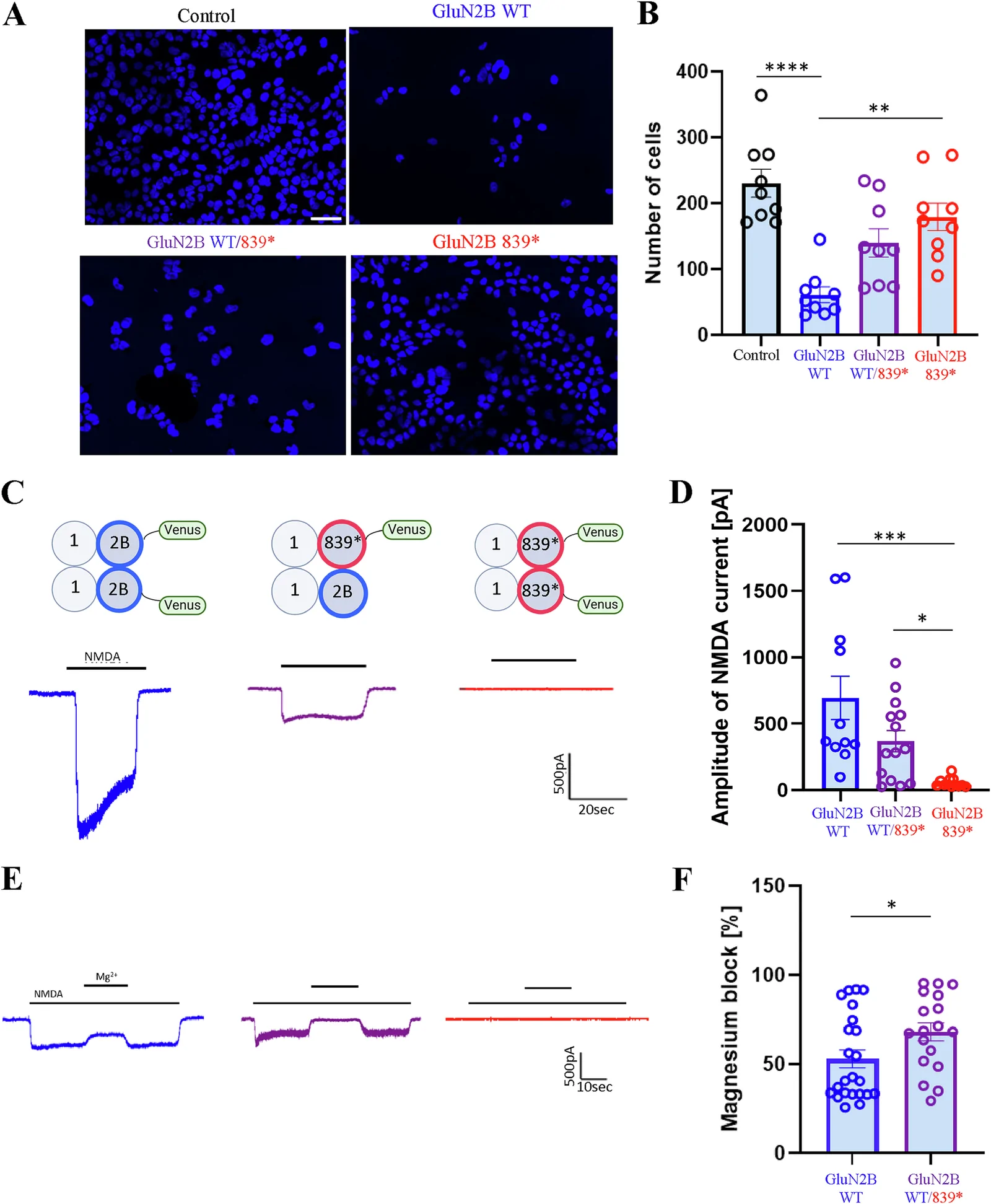
GluN2B CTD deletion hinders NMDA-R function. **(A)** and **(B)** WT- but not GluN2B-E839*-containing NMDA-R expression reduces cell number. Representative illustration (A) and quantification (B) of Hoechst nucleus staining showing the total number of cells per field of view 24 h after transfection; scale bar: 50 μm. (B) Bar graphs are mean ± SEM of the number of cells transfected with an empty vector (control) or plasmids coding for GluN1 and GluN2B (WT), GluN2B/GluN2B-E839* (WT/839*) or GluN2B-E839* (839*). Kruskal-Wallis test p < 0.0001, with Dunn’s multiple comparisons: **, *p*-value < 0.01; ****, *p*-value < 0.0001. Results were analyzed on a pooled data set from 3 independent experiments. **(C-F)** GluN2B CTD deletion decreases NMDA-induced currents. Scheme of constructs and representative whole-cell currents (C and E), and quantifications (D and F), evoked by rapid application of 100 μM NMDA in the presence of 10 μM glycine, with (E and F) or without (C and D) 1 mM magnesium (Mg^2+^). Cells were clamped at −60 mV. (D) Bar graphs are mean ± SEM of peak currents. Kruskal-Wallis test p < 0.0001, with Dunn’s multiple comparisons: *, *p*-value < 0.05; ***, *p*-value < 0.0001. (F) Bar graphs are mean ± SEM of the percentage of the NMDA current blocked by 1 mM Mg^2+^; Mann-Whitney test; *, p < 0.05 (GraphPad Prism 7).

Excessive activation of NMDA-Rs can lead to cytotoxicity due to an influx of calcium ions, ultimately causing neuronal damage and cell death. Consistently, the number of cells reported by Hoechst nuclear staining (Fig. 5A and B) was significantly reduced in HEK293T cell cultures transfected with the wild-type NMDA-R compared to non-transfected cells. In contrast, the expression of GluN2B-E839*-containing NMDA-Rs does not significantly impact cell number in the homozygous state compared to untransfected cells, while the heterozygous state results in an intermediate cell loss, in between cultures expressing wild-type NMDA-R and those expressing GluN2B-E839*-containing NMDA-Rs only. Of note, these differences are unlikely to result from variations in transfection efficiency, as identical transfection conditions were used across all experiments and consistently yielded robust receptor expression under all conditions (Fig. 3B). These findings show a detrimental, excitotoxic, effect of the GluN2B WT but not of the mutant GluN2B-E839* on cell viability, which could be attributed to the dysfunction of the mutated receptor.

To directly assess NMDA-R functioning, we measured NMDA currents using whole-cell patch clamp recordings in HEK293T cells co-expressing GluN1 with GluN2B WT (WT condition), GluN1 with both GluN2B WT and GluN2B-E839* variant (heterozygous) or GluN1 with GluN2B-E839* variant (homozygous model, Fig. 5C). Inward currents evoked by rapid application of 100 μM NMDA were significantly higher in cells expressing wild-type GluN2B (mean amplitude of 693.591 ± 165.198 pA) compared to homozygous GluN2B-E839* condition (54.392 ± 10.345 pA, Fig. 5D), while in the heterologous condition cells co-expressing GluN2B WT and GluN2B-E839* subunits displayed intermediate NMDA-induced currents amplitude (368.579 ± 80.676 pA). NMDA-R composed of two GluN2B-E839* subunits was barely not functional. The very weak amount of NMDA currents recorded in NMDA-Rs containing only mutated GluN2B-E839*subunits, hampered quantifying the effect of Mg^2+^ 1 mM in the homozygous condition. However, in the heterozygous condition, we measured a significant increase in the magnesium block of NMDA-Rs compared to the WT receptors (Fig. 5E and F). Overall, a GluN2B-E839*-containing receptor exhibits not only reduced inward currents but also increased sensitivity to magnesium inhibition. Results from the heterologous expression system suggest that GluN2B-E839* variant may lead to dysfunction in patient cells.

### Assessment of GluN2B-E839* induced cellular dysfunction in patient iPSC-derived neurons

To characterize cellular perturbations induced by the GluN2B-E839* variant, we used neurons derived from patient iPSCs. This model involves differentiating neurons from pluripotent cells reprogrammed from the GluN2B-E839* patient fibroblasts, alongside control ones from a healthy donor. This human preclinical model preserves the patient’s genetic background and disease-causing mutations, making it crucial for understanding genetic contributions to disease.

#### Characterization of cortical neurons derived from patient iPSCs

**Fig. 6:**
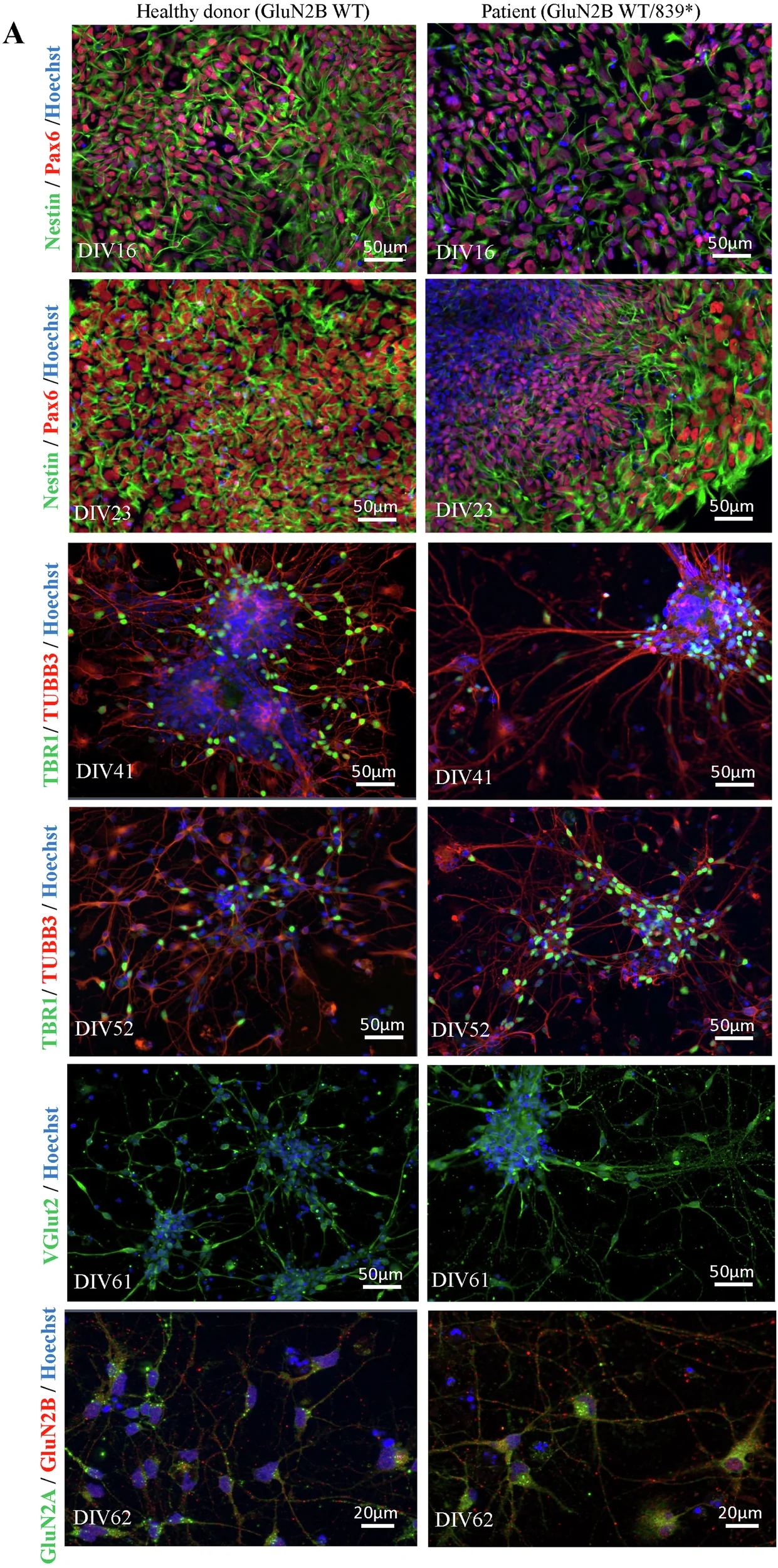

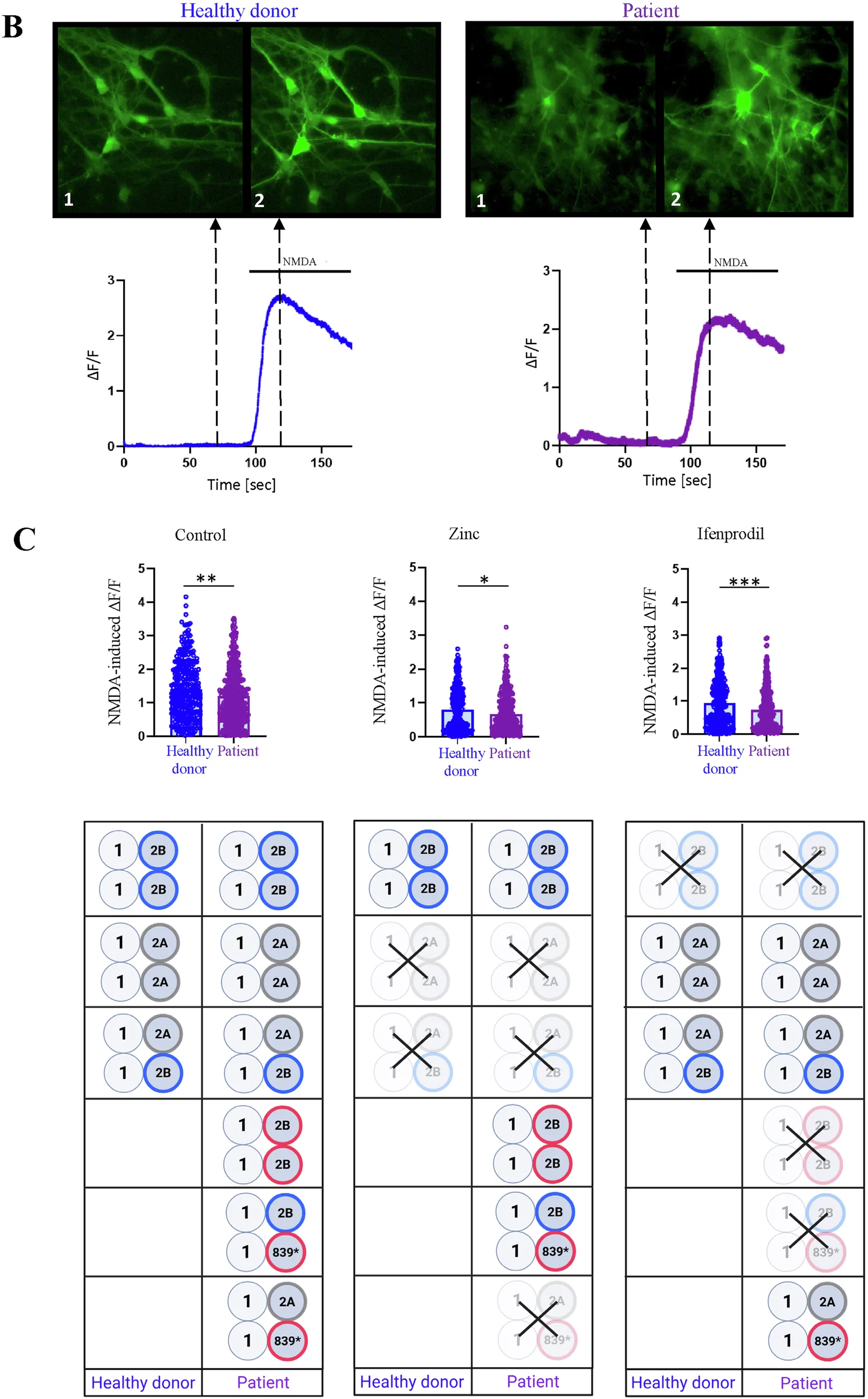
Impaired calcium signaling in cortical neurons derived from GluN2B-E839* hiPSC. **A** Immunofluorescent staining of Nestin and Pax6 at Day In Vitro 16 (DIV16) and DIV23, TBR1 and TUBB3 at DIV41 and DIV52, VGlut2 at DIV61, GluN2A and GluN2B at DIV62, in control’s (left column) and patient’s (right column) human iPS-cell derived neurons. **B** Representative illustrations of intracellular calcium fluctuations reported by GCaMP6s fluorescence variations, in human iPSCs-derived neurons continuously perfused using a fast gravity perfusion system, before and during NMDA (100 µM) application. **C** Bar graph representing mean ± SEM NMDA-induced ΔF/F in absence (control) or in presence of zinc (50 nM) or ifenprodil (100 nM) in the bath. Mann-Whitney test; *, *p*-value ≤ 0.05; **, *p*-value ≤ 0.01; *** *p* ≤ 0,001 (GraphPad Prism 7). Below graphs, the nature of NMDA-R subunits through which calcium influx was recorded in each condition is represented.

Following the isolation of human dermal fibroblasts, we generated iPSC lines, which we classified and validated for pluripotency and genetic stability (Supp. Fig. S2). Next, control and GluN2B-E839* hiPSCs were differentiated into cortical neurons [15]. We confirmed the identity and maturation level of iPSC-derived cortical neurons by immunochemistry (Fig. 6A). Sixteen days after neural induction (Days In Vitro, DIV16) human iPSCs mostly differentiated into Nestin- and PAX6-positive neural progenitors. These early-stage neuroectodermal markers suggested that the iPSC-derived compact colony-forming cells were early-stage neural stem cells (NSCs). After DIV40, we observed the presence of neurons (positive for βIII-tubulin (TUBB3)), expressing the forebrain pyramidal cell marker – TBR1. The proportion of TBR1-positive neurons kept increasing at DIV52. Around DIV60, pyramidal-shaped neurons expressed vesicular glutamate transporters 2 (VGLUT2), indicating successful differentiation into cortical glutamatergic neurons. We further characterized post-synaptic markers by the expression of GluN2A and 2B subunits of NMDA-Rs. The existence of NMDA-Rs on the neuronal cell bodies was also indicative of terminal differentiation and suggested functional and physiological maturity. Comparable differentiation efficiency was observed across all cell lines, suggesting that the GluN2B-E839* variant does not affect neuronal differentiation in vitro. Spontaneous and evoked activities recorded by the current clamp technique further validated the maturation and functionality of pyramidal-shaped neurons, from patient and control, after DIV60 (Supp. Fig. S2G and H). Human iPSC-generated neurons were electrophysiologically active and formed functional synapses.

#### The GluN2B-E839* variant alters Ca^2+^ influx

Due to their calcium permeability, NMDA-Rs play a critical role in physiological signaling and neuronal plasticity. We therefore studied whether the molecular dysfunctions caused by the GluN2B-E839* variant had an impact on human neurons signaling, through live cell imaging of GCaMP6s calcium sensor fluorescence. Ca^2+^ fluctuations from regions of interest (ROIs) were reported as the relative fluorescence changes ΔF/F_0_. In both types of neurons – derived from GluN2B-E839* and control patients iPSCs, we measured spontaneous Ca^2+^ transients. Using a fast gravity perfusion system, NMDA (100 μM) application induced a massive increase in intracellular Ca^2+^ for both control individuals and GluN2B-E839* patient neurons (Fig. 6B). The GluN2B-E839* variant however significantly reduced the amplitude of NMDA-induced calcium influx (ΔF/F_0_ = 1.204 ± 0.041) compared to control neurons (ΔF/F_0_ = 1.401 ± 0.049). Using pharmacology, we further specifically assessed the functionality of NMDA-Rs, depending on their subunit composition [19]. Zinc displays a higher affinity for GluN2A subunit-containing receptors and is considered as a specific inhibitor at 50 nM. Preventing the influx of calcium through GluN2A subunits-containing receptors enabled focusing on GluN1/GluN2B diheteromers (Fig. 6C). As expected, in presence of zinc (50 nM), the NMDA-induced calcium influx was significantly reduced in neurons derived from GluN2B-E839* patient iPSCs (ΔF/F_0_ = 0.677 ± 0.027) compared to the control ones (ΔF/F_0_ = 0.813 ± 0.036), confirming a reduction of calcium influx through GluN1/GluN2B diheteromers caused by the GluN2B-E839* variant. Reversely, we used ifenprodil (100 nM), a selective GluN1/GluN2B diheteromers antagonist, to isolate calcium influx from all other NMDA-Rs (Fig. 6C). In presence of ifenprodil, the NMDA-induced Ca^2+^ influx was still significantly reduced in GluN2B-E839* neurons (ΔF/F_0_ = 0.747 ± 0.032) compared to control (ΔF/F_0_ = 0.955 ± 0.041). The residual difference between genotypes may arise from receptors composed of GluN1/GluN2A/GluN2B in control patient neurons and GluN1/GluN2A/GluN2B-E839* in neurons derived from the GluN2B-E839* patient, consistent with the predominance of these triheteromeric receptors. [4, 19]. We cannot exclude the possibility that, under Ifenprodil conditions, genotype differences may also result from a neurodevelopmental adaptation in GluN2A subunit expression in response to GluN2B mutation.

Taken together, the calcium measurement data from patient-derived neurons confirm NMDA-R dysfunction in the human preclinical model and suggest potential deficits in neuronal plasticity in DEEs patients.

## Discussion

This study provides a comprehensive functional characterization of a pathogenic truncating *GRIN2B* variant, GluN2B-E839*, and offers mechanistic insight into how the loss of the GluN2B CTD perturbs NMDA receptor expression, trafficking, and signaling in the context of DEEs. While pathogenic *GRIN* variants are distributed across all receptor domains, functional studies have predominantly focused on missense variants affecting structured regions such as the ligand-binding and transmembrane domains, which directly alter agonist affinity, channel gating, ion permeation, or voltage-dependent Mg^2+^ block. In contrast, truncating variants affecting the intrinsically disordered CTD remain comparatively underexplored [20, 21], despite representing a substantial fraction of *GRIN2B* variants identified in patients.

By combining heterologous expression systems with patient-derived iPSC cortical neurons, we show that GluN2B-E839* assembles efficiently with GluN1 and can co-exist with wild-type GluN2B within the same receptor complexes, consistent with the heterozygous condition identified in the patient. However, receptors containing the truncated subunit exhibit markedly reduced surface trafficking, impaired interaction with the postsynaptic scaffold PSD-95, diminished NMDA-evoked currents, and increased sensitivity to voltage-dependent Mg^2+^ block. In patient-derived neurons, these molecular defects translate into reduced NMDA receptor–mediated Ca^2+^ influx, indicating that CTD loss has functionally relevant consequences in a human neuronal context. Together, these findings support a dominant-negative effect of the truncation rather than simple haploinsufficiency, primarily driven by altered trafficking, synaptic stabilization, and intracellular signaling.

Importantly, the mechanistic consequences of CTD truncation differ fundamentally from those associated with variants affecting other receptor domains. Whereas missense mutations in the ligand-binding or transmembrane regions typically modify channel gating, agonist potency, or ion conductance, CTD truncations disrupt processes downstream of receptor assembly, including surface delivery, synaptic anchoring, and coupling to intracellular signaling pathways [6, 20, 21]. Although Mg^2+^ block is classically attributed to residues within the M2 pore loop, our observation that CTD loss indirectly increases Mg^2+^ sensitivity suggests the existence of long-range allosteric coupling between intracellular domains and the transmembrane pore. This mechanism remains poorly understood and highlights the need to consider intracellular domains as active modulators of core receptor biophysics.

These findings align with recent reports showing that disease-associated nonsense and frameshift variants truncating the GluN2A and GluN2B CTD reduce NMDA receptor surface expression and synaptic localization in heterologous systems [22], as well as in a transgenic mouse models expressing CTD-truncated GluN2B subunits [23]. In addition, while our study was ongoing, the GluN2B-E839* variant was independently reported in the Human Gene Mutation Database (HGMD®), associated with intellectual disability (CM211520), and shown to impair receptor surface expression and current amplitude in heterologous systems [24]. However, our study extends this finding by linking CTD loss to altered receptor function and Ca^2+^ signaling in patient-derived neurons, thereby strengthening the translational relevance of CTD dysfunctions in GRIN2B-related disorders.

Recent findings on rare or de novo variants in *GRIN* genes, including those encoding the GluN2B subunit of NMDA-Rs, have emphasized the importance of linking genetic variation to structural and functional receptor dysfunctions. Advances in the characterization of *GRIN* variants have largely focused on receptor-level properties such as agonist and co-agonist binding, channel gating, voltage-dependent Mg^2+^ block, and surface expression, primarily using recombinant receptors expressed in heterologous systems, and increasingly complemented by neuronal and in vivo models [6]. In this context, recent work from the Traynelis and Yuan groups has proposed a comprehensive and clinically oriented framework to classify *GRIN* missense variants along a gain- versus loss-of-function (GoF/LoF) axis by integrating multiple functional readouts [25] This approach responds to a pressing clinical need, as GRIN-related disorders are individually rare but collectively frequent, requiring rapid functional stratification to inform diagnosis, personalized therapeutic strategies, and clinical trial design. Complementary structure-informed computational approaches further support this effort by enabling scalable prediction of variant pathogenicity and functional direction, notably through spatial relationships to ligands or the pore axis and conserved structural features across ion channels [26, 27] Our results underscore both the utility and the limitations of such simplified classification schemes when applied to truncating variants affecting intrinsically disordered regions. While CTD truncations may ultimately manifest as functional loss at the synaptic level, the underlying molecular mechanisms differ substantially from those of variants that directly impair channel function, emphasizing the importance of domain-specific mechanistic interpretation.

From a methodological perspective, the use of both heterologous systems and patient-derived neurons was essential to capture the multifaceted consequences of CTD loss. Heterologous expression allowed precise control of subunit composition and quantitative assessment of assembly, trafficking, and biophysical properties, whereas iPSC-derived neurons preserved the endogenous subunit diversity and cellular context necessary to evaluate disease-relevant signaling. Our use of a non-isogenic healthy control may represent a limitation as genetic background variability may influence cellular phenotypes. Although isogenic correction would provide a powerful means to strengthen causal inference, such an approach is technically challenging for this variant and may introduce additional sources of variability related to genome editing and clonal selection. To mitigate these issues, control and patient fibroblasts were obtained using identical procedures and reprogrammed and differentiated in parallel. In this context, the unrelated healthy control provides an important benchmark for normal biological variation. Notably, NMDA-evoked Ca^2+^ influx in patient-derived neurons reflects the combined activity of multiple endogenously expressed NMDA receptor subtypes. Pharmacological dissection revealed that the GluN2B-E839* variant (compared to healthy donor) affects not only GluN1/GluN2B diheteromers but also triheteromeric GluN1/GluN2A/GluN2B receptors, indicating that CTD loss has broad functional consequences beyond a single receptor population. This complexity highlights both the strength and the inherent limitations of human neuronal models, which more closely reflect the in vivo condition but complicate direct attribution of effects to individual receptor assemblies.

From a translational perspective, these distinctions are critical. A binary loss-of-function classification might suggest pharmacological enhancement of NMDA receptor activity; however, such an approach may be ineffective or inappropriate when the primary defect lies in receptor trafficking or synaptic localization rather than channel conductance. Our findings therefore emphasize the need for fine-grained mechanistic characterization of GRIN variants to guide rational therapeutic strategies and avoid interventions that fail to target the underlying molecular dysfunction. While NMDA receptor modulators hold promise, their use must be carefully balanced against potential risks in neurodevelopmental disorders. In summary, by integrating patient genetics, clinical features, and multi-level functional analyses, our work, applicable to any *GRIN* gene variant, contributes to establishing a more nuanced framework for variant interpretation and highlights the importance of considering domain-specific mechanisms when developing precision therapies for GRIN-related disorders.

## Supplementary information

Supplemental material

## Data Availability

All data are available in the main text or the supplementary materials.
