# Supplementary material for: Clinic-to-Mechanism: Unraveling in-depth molecular dysfunctions caused by a GluN2B C-Terminal deletion in developmental and epileptic encephalopathies
Source: Transl Psychiatry. 2026 Jun 24;16:457. doi: 10.1038/s41398-026-04186-0 (PMC13550643; doi:10.1038/s41398-026-04186-0)
Supplement: Supplementary file 1 [file 41398_2026_4186_MOESM1_ESM.docx]

**Supplementary Materials**

Supplementary data

*Isolation of Human Dermal Fibroblasts and Generation of iPSC Lines*

To determine the consequences of GluN2B-E839* variant in a human model, we isolated skin fibroblasts from the patient carrying the GluN2B-E839* variant (NM_000834.5; c.2515G>T; p.Glu839Ter). Then, we transfected dermal fibroblasts with a mixture of 6 mRNA pluripotent-specific transcription factors (Oct4, Klf4, Sox2, Lin28, c-Myc, Nanog), reporter proteins (nGFP, eGFP) and miRNA. Following the reprogramming, we confirmed the presence of original mutation in diagnostic PCR analysis in all iPSC lines generated from the patient. All iPSC lines were heterozygous for the mutation.

*Classification and Validation of iPSC Lines Generated from Donor*

Based on representative iPSC morphology and growth rate, we selected two patient and two healthy donor iPSC lines for further validation analysis (Supp. Fig. S2). Karyotype and copy number variation (CNV) were verified for all the clones. All iPSC lines expressed endogenous pluripotency markers OCT4, SOX2, and TRA1-81 (immunocytochemistry from Supp. Fig. S2E). We also tested the differentiation potential of the converted iPSC lines into the three germ layer markers using STEMdiff™ Trilineage Differentiation Kit (Supp. Fig. S2E). In summary, all iPSC lines were validated for pluripotency markers, differentiation potential, and the absence of chromosomal aberrations.

*Generation of progenitors and neurons*

We used the iPSC line carrying the GluN2B-E839* variant to generate progenitors and cortical neurons. The neuronal differentiation of mutated cells was compared to healthy donor. The Supp. Fig. S2F indicates the morphological progression of the iPSC-cortical neuronal cultures. Around DIV21, cells forming typical neural tube-like rosettes were observed. From DIV40, axonal and dendritic extensions started to appear. Neurons with extensive neurites were readily present in all cultures of GluN2B-E839*and healthy donor cell lines. From DIV40 to DIV62, significant extension of neuronal processes was noted, along with the presence of network-like morphology. Comparable differentiation efficiency was observed across all cell lines.

Supplementary Table

**Supplementary Table S1 - List of antibodies used for immunofluorescence and Western blot analysis**

**Supplementary Table S2 - Summary of statistical tests**

**Supplementary Table S3 - Description of 48 genes included in panel EIEE v.1 / 2016**

Supplementary Figures

**Supplementary Fig. S1 - Study design**

Study design including tNGS analysis, selection of mutation in *GRIN2B* gene and functional studies using two cellular models - transfected HEK cells and iPSC-derived neurons.

**Supplementary Fig. S2 - Characterization of iPSCs pluripotency and ability to differentiate - example of a healthy donor and patient cell line.**

**(A)** Table containing key information about the healthy donor and patient cell line **(B)** Human iPSCs at days 1-4 after passage. Images were taken using a magnification of 5X. For this culture, day 4 was the optimal time for passaging. **(C)** Exclusion of chromosomal aberration by chromosome analysis. **(D)** Analysis of copy number using Stem Genomics iPCS-digital™ PSC 24 probe kit. The results show no CNV abnormality. **(E)** Immunofluorescent staining of the pluripotency markers - nuclear stem cell markers OCT4 and surface markers SSEA-4 and TRA-1-81 and specific markers for early differentiation for ectoderm overlay with a staining of the nucleus with Hoechst (scale bar 50 μm). **(F)** Overview of the neuronal differentiation protocol and representative images at different stages. Scale bar = 50 μm. Images taken using a magnification of x10 (1-14DIV), x5 (DIV21) and x32 (41-61DIV). **(G)** and **(H)** Representative traces of spontaneous (G) and evoked (H) action potentials (APs) from current-clamp recordings in control iPSC-derived neurons at DIV60. **(I)** No difference (Z-test for proportions) could be observed in neurons derived from healthy donor (Ctrl, n=13) and patient (E839*, n=8) iPSCs regarding the proportion of cells showing voltage-gated sodium current (INa, upper graphs, p=0.78), the proportion of cells with spontaneous activity (middle graphs, p=0.75), and the proportion of cells displaying evoked activity (lower graphs, p=0.70).
